# Supplementary material for: Trophic niches of a seabird assemblage in Bass Strait, south-eastern Australia
Source: PeerJ. 2020 Mar 11;8:e8700. doi: 10.7717/peerj.8700 (PMC7071825; doi:10.7717/peerj.8700)

**Trophic niches in a seabird assemblage in Bass Strait, south-eastern Australia**

Aymeric Fromant^1,2^, Nicole Schumann^1^, Peter Dann^3^, Yves Cherel^2^ and John P.Y. Arnould^1^

^1^ School of Life and Environmental Sciences, Deakin University, 221 Burwood Hwy, Burwood, VIC 3125, Australia

^2^ Centre d’Etudes Biologiques de Chizé (CEBC), UMR 7372 du CNRS – La Rochelle Université, 79360 Villiers en Bois, France

^3^ Research Department, Phillip Island Nature Parks, PO Box 97, Cowes, VIC 3922, Australia

**SUPPLEMENTARY TEXT**

Three Layman metrics were used as a measure of niche variation between the species, region and years (Layman et al. 2007):

**1) *δ*^13^C range (CR):** Distance between the most enriched and most depleted ^13^C values (i.e., maximum *δ*^13^C - minimum *δ*^13^C). Increased CR would be expected if there are multiple basal resources with varying *δ*^13^C values;

**2) *δ*^15^N Range (NR):** Distance between the most enriched and most depleted ^15^N values (i.e., maximum *δ*^15^N - minimum *δ*^15^N). NR is a representation of vertical structure. A larger range in *δ*^15^N among consumers suggests more trophic levels and thus a greater degree of trophic diversity;

**3) Total area (TA):** Convex hull area encompassed by all samples in *δ*^13^C– *δ*^15^N bi-plot space. This represents a measure of the total amount of niche space occupied, and thus a proxy for the total extent of trophic diversity within this group.

**References:**

Layman CA, Arrington DA, Montaña CG, Post DM (2007) Can stable isotope ratios provide for community-wide measures of trophic structure? Ecology 88:42-48

**Table S1:**

**Frequency of occurrence (FO) and mean numerical abundance (NA) per sample ± SE of taxa recovered from little penguins stomach samples in winter 2008 (n = 20) and summer 2009 (n = 22) in central Bass Strait.**

| **Prey taxa** | **August 2008 (*n* = 20)** | | | **January 2009 (*n* = 22)** | | |
| --- | --- | --- | --- | --- | --- | --- |
|  |  | **FO** | **NA** |  | **FO** | **NA** |
|  | ***n*** | **%** | **%** | ***n*** | **%** | **%** |
| **Fish** | **20** | **100** | **79.7** | **22** | **100** | **68.8** |
| Jack mackerel *Trachurus declivis* | 19 | 95 | 69.1 | 1 | 4.5 | 0.3 |
| Pilchard *Sardinops sagax* | 5 | 25 | 1.5 | 11 | 50 | 6.1 |
| Southern sea garfish *Hyporhamphus melanochir* | 4 | 20 | 1.5 | - | - | - |
| Australian anchovy *Engraulis australis* | 3 | 15 | 0.9 | 19 | 86.4 | 32.2 |
| Silverbelly *Parequula melbournensis* | 1 | 5 | 0.3 | - | - | - |
| Unidentified postlarval fish | - | - | - | 3 | 13.6 | 23.2 |
| Unidentified postlarval pipefish/seahorse | - | - | - | 1 | 4.5 | 0.6 |
| Barracouta *Thyrsites atun* | - | - | - | 5 | 22.7 | 1.9 |
| Blue sprat *Spratelloides robustus* | - | - | - | 1 | 4.5 | 0.3 |
| Unidentified pilchard/anchovy | - | - | - | 1 | 4.5 | 0.3 |
| Unidentified gemfish/barracouta | - | - | - | 1 | 4.5 | 0.3 |
| Unidentified fish | 8 | 40 | 6.4 | 8 | 36.4 | 3.5 |
| **Crustaceans** | **13** | **65** | **18.2** | **13** | **59.1** | **23.5** |
| Cymothoid  Isopod *Ceratothoa imbricata* | 12 | 60 | 17.9 | - | - | - |
| Cirolanid Isopod *Eurydice tarti* | - | - | - | 4 | 18.2 | 3.2 |
| Amphipod sp. | - | - | - | 2 | 9.1 | 0.6 |
| Copepod sp. | - | - | - | 1 | 4.5 | 0.3 |
| Unidentified crustaceans | 1 | 5 | 0.3 | 13 | 59.1 | 19.3 |
| **Cephalopods** | **6** | **30** | **2.1** | **4** | **18.2** | **7.7** |
| Gould's squid *Nototodarus gouldi* | 6 | 30 | 2.1 | 3 | 13.6 | 1 |
| Unidentified cephalopods | - | - | - | 1 | 4.5 | 6.6 |

**Table S2:**

**Frequency of occurrence (FO) and mean numerical abundance (NA) per sample ± SE of taxa recovered from short-tailed shearwaters stomach samples in summer 2009 (n = 25) and 2010 (n = 26) in central Bass Strait.**

| **Prey taxa** | **January 2009 (*n* = 25)** | | |  | **January 2010 (*n* = 26)** | | |
| --- | --- | --- | --- | --- | --- | --- | --- |
|  |  | **FO** | **NA** |  |  | **FO** | **NA** |
|  | ***n*** | **%** | **%** |  | ***n*** | **%** | **%** |
| **Fish** | **10** | **40.0** | **2.6** |  | **12** | **46.2** | **1.0** |
| Post-larval fish | 10 | 40.0 | 2.6 |  | 11 | 42.3 | 1.0 |
| Blue warehou *Seriolella brama* | - | - | - |  | 1 | 3.8 | > 0.01 |
| Unidentified adult fish | - | - | - |  | 3 | 11.5 | 0.02 |
| **Crustaceans** | **24** | **96.0** | **97.2** |  | **26** | **100** | **98.8** |
| Coastal krill *Nyctiphanes australis* | 19 | 76.0 | 78.6 |  | 26 | 100 | 98.2 |
| *Euphausia* sp. | 2 | 8.0 | 10.2 |  | - | - | - |
| Hyperiid amphipod *Themisto australis* | 5 | 20.0 | 8.0 |  | 3 | 11.5 | 0.1 |
| Hyperiid amphipod *Themisto gaudichaudii* | 2 | 8.0 | 0.1 |  | - | - | - |
| Copepod sp. | 1 | 4.0 | 0.1 |  | 1 | 3.8 | > 0.01 |
| Juvenile isopod | 1 | 4.0 | 0.03 |  | - | - | - |
| Swimming crab megalopa *Ovalipes* sp. | - | - | - |  | 5 | 19.2 | 0.1 |
| Unidentified stomatopod larvae | - | - | - |  | 1 | 3.8 | > 0.01 |
| Unidentified crustaceans | 5 | 20.0 | 0.2 |  | 1 | 3.8 | 0.4 |
| **Cephalopods** | **5** | **20.0** | **0.2** |  | **4** | **15.4** | **0.1** |
| Unidentified squid | 5 | 20.0 | 0.2 |  | 2 | 7.7 | 0.01 |
| Gould's squid *Nototodarus gouldi* | - | - | - |  | 1 | 3.8 | 0.02 |
| Unidentified cephalopod | - | - | - |  | 2 | 7.7 | 0.04 |

**Table S3:**

**Frequency of occurrence (FO) and mean numerical abundance (NA) per sample ± SE of taxa recovered from stomach samples of common diving petrels in winter 2008 (n = 3) and 2009 (n = 3) and fairy prions in summer 2011 (n = 20) in central Bass Strait.** Due to the small sample size for common diving petrels (respectively 3 individuals in 2008 and 3 individuals in 2009), the data of both years were combined (no interannual difference).

| **Prey taxa** | **Fairy prion** | | |  |  | **Common diving-petrel** | |
| --- | --- | --- | --- | --- | --- | --- | --- |
|  | **January 2011 (*n* = 20)** | | |  |  | **July 2008 (*n* = 3) - 2009 (*n* = 3)** | |
|  |  | **FO** | **NA** |  |  | **FO** | **NA** |
|  | ***n*** | **%** | **%** |  | ***n*** | **%** | **%** |
| **Fish** | **5** | **25** | **0.03** |  | - | - | - |
| Post-larval fish | 2 | 10 | 0.01 |  | - | - | - |
| Cod *Pseudophycis* sp. | 1 | 5 | 0.01 |  | - | - | - |
| Unidentified adult fish | 2 | 10 | 0.01 |  | - | - | - |
| **Crustaceans** | **20** | **100** | **99.97** |  | **6** | **100** | **100** |
| Coastal krill *Nyctiphanes australis* | 20 | 100 | 93.5 |  | 4 | 66.7 | 91.6 |
| Synopiid amphipod *Telsosynopia trifidilla* | 4 | 20 | 0.02 |  | - | - | - |
| Swimming crab megalopa *Ovalipes* sp. | 20 | 100 | 5.9 |  | - | - | - |
| Unidentified stomatopod larvae | 13 | 65 | 0.5 |  | - | - | - |
| Hyperiid amphipod sp. | 6 | 30 | 0.03 |  | 2 | 33.3 | 4.2 |
| Unidentified amphipod sp. | 2 | 10 | 0.01 |  | - | - | - |

**Table S4:**

**Results of the GLM models explaining stable isotope values (*δ^15^*N and *δ^13^*C) for little penguins (n = 137), short-tailed shearwaters (n = 167), fairy prions (n = 59) and common diving petrels (n = 36).**

Explanatory variables, deviance explained, Akaike Information Criterion (AIC) and Delta AIC (difference in AIC with the best model) are given for each model. Models are ranked according to their respective AIC value and significant variables in each model are highlighted in bold.

|  | Model | Deviance | AIC | Delta AIC |
| --- | --- | --- | --- | --- |
| Little penguin  (summer + winter) | *δ^15^*N ~ ***δ^13^*C** * **REGION** * **YEAR*SEASON** | 80.6 % | 342.5 | 0.0 |
|  | *δ^15^*N ~ *δ^13^*C + **REGION** + **YEAR +** SEASON | 39.4 % | 568.0 | 225.5 |
|  | *δ^15^*N ~ **REGION** | 23.9 % | 613.7 | 271.2 |
|  | *δ^15^*N ~ **YEAR** | 14.6 % | 644.0 | 301.5 |
|  | *δ^15^*N ~ *δ^13^*C | 3.1 % | 670.8 | 328.3 |
|  | *δ^15^*N ~ 1 | 0.0 % | 676.5 | 334 |
|  | *δ^15^*N ~ SEASON | 0.2 % | 678.0 | 335.5 |
|  | *δ^13^*C ~ **REGION** * **YEAR*SEASON** | 70.3 % | 90.9 | 0.0 |
|  | *δ^13^*C ~ **REGION** + **YEAR +** SEASON | 54.4 % | 175.7 | 84.8 |
|  | *δ^13^*C ~ **REGION** | 42.6 % | 223.4 | 132.6 |
|  | *δ^13^*C ~ **YEAR** | 8.4 % | 339.8 | 248.9 |
|  | *δ^13^*C ~ 1 | 0.0 % | 355.1 | 264.2 |
|  | *δ^13^*C ~ SEASON | 0.4 % | 356.0 | 265.1 |
| Short-tailed shearwater  (summer) | *δ^15^*N ~ ***δ^13^*C** * **REGION** * **YEAR** | 71.2 % | 217.5 | 0.0 |
|  | *δ^15^*N ~ ***δ^13^*C** + REGION + **YEAR** | 54.5 % | 269.7 | 52.2 |
|  | *δ^15^*N ~ **YEAR** | 43.5 % | 299.8 | 82.3 |
|  | *δ^15^*N ~ ***δ^13^*C** | 32.3 % | 328.0 | 110.5 |
|  | *δ^15^*N ~ 1 | 0.0 % | 391.2 | 173.7 |
|  | *δ^15^*N ~ REGION | 0.1 % | 395.0 | 177.5 |
|  | *δ^13^*C ~ **REGION** * **YEAR** | 66.4 % | 165.7 | 0.0 |
|  | *δ^13^*C ~ **REGION** + **YEAR** | 28.4 % | 284.1 | 118.4 |
|  | *δ^13^*C ~ **YEAR** | 25.4 % | 287.0 | 121.3 |
|  | *δ^13^*C ~ 1 | 0.0 % | 331.9 | 166.2 |
|  | *δ^13^*C ~ REGION | 2.3 % | 332.0 | 166.3 |
| Fairy prion  (summer + winter) | *δ^15^*N ~ ***δ^13^*C** * **REGION** * **YEAR * SEASON** | 59.9 % | 224.6 | 0.0 |
|  | *δ^15^*N ~ **SEASON** | 27.1 % | 247.7 | 23.1 |
|  | *δ^15^*N ~ *δ^13^*C + REGION + **YEAR + SEASON** | 33.5 % | 248.2 | 23.6 |
|  | *δ^15^*N ~ **YEAR** | 17.1 % | 260.2 | 35.6 |
|  | *δ^15^*N ~ 1 | 0.0 % | 271.7 | 47.1 |
|  | *δ^15^*N ~ REGION | 1.1 % | 272.8 | 48.2 |
|  | *δ^15^*N ~ *δ^13^*C | 0.0 % | 273.6 | 48.6 |
|  | *δ^13^*C ~ **REGION** * **YEAR * SEASON** | 54.7 % | 110.3 | 0.0 |
|  | *δ^13^*C ~ **REGION** + YEAR + SEASON | 51.4 % | 112.0 | 1.7 |
|  | *δ^13^*C ~ **REGION** | 37 % | 127.3 | 17.0 |
|  | *δ^13^*C ~ SEASON | 2.6 % | 163.0 | 52.7 |
|  | *δ^13^*C ~ 1 | 0.0 % | 163.2 | 52.9 |
|  | *δ^13^*C ~ YEAR | 2.3 % | 165.3 | 55.0 |
| Common diving petrel  (winter) | *δ^15^*N ~ ***δ^13^*C** * **REGION** * **YEAR** | 92.6 % | 3.0 | 0.0 |
|  | *δ^15^*N ~ ***δ^13^*C** + **REGION** + **YEAR** | 92.6 % | 8.8 | 5.8 |
|  | *δ^15^*N ~ **YEAR** | 85.0 % | 24.4 | 21.4 |
|  | *δ^15^*N ~ **REGION** | 23.1 % | 81.2 | 78.2 |
|  | *δ^15^*N ~ ***δ^13^*C** | 17.5 % | 83.8 | 80.8 |
|  | *δ^15^*N ~ 1 | 0.0 % | 88.7 | 85.7 |
|  | *δ^13^*C ~ **REGION** * **YEAR** | 58.6 % | 5.1 | 0.0 |
|  | *δ^13^*C ~ **REGION** + YEAR | 58.6 % | 5.1 | 0.0 |
|  | *δ^13^*C ~ **REGION** | 58.3 % | 9.6 | 4.5 |
|  | *δ^13^*C ~ 1 | 0.0 % | 63.8 | 58.3 |
|  | *δ^13^*C ~ YEAR | 7.3 % | 66.4 | 60.9 |

**Table S5:**

**Degree of niche overlap (%) among species for each region, year and season.**

Each table combined the data for one region and one season. The niche overlap was calculated between the 4 study species for the same year of sampling, or between one study species only among the different sampled years.

| **Western Bass Strait summer** | | | | | | | | | | | | |
| --- | --- | --- | --- | --- | --- | --- | --- | --- | --- | --- | --- | --- |
|  |  | **2009** | | | **2010** | | | | **2011** | | | |
|  |  | LP  (n = 10) | STSW  (n = 16) | FP  (n = 8) | LP  (n = 20) | STSW  (n = 20) | | FP  (n = 10) | LP  (n = 20) | STSW  (n = 20) | FP  (n = 18) | |
| **2009** | LP (n = 10) |  | 0 | 4.8 | 3.7 | - | | - | 36.0 | - | - | |
|  | STSW (n = 16) |  |  | 0 | - | 14.8 | | - | - | 53.7 | - | |
|  | FP (n = 18) |  |  |  | - | - | | 22.7 | - | - | 16.8 | |
| **2010** | LP (n = 20) |  |  |  |  | 0 | | 15.8 | 15.1 | - | - | |
|  | STSW (n = 20) |  |  |  |  |  | | 0 | - | 7.3 | - | |
|  | FP (n = 10) |  |  |  |  |  | |  | - | - | 38.3 | |
| **2011** | LP (n = 20) |  |  |  |  |  | |  |  | 0 | 3.8 | |
|  | STSW (n = 20) |  |  |  |  |  | |  |  |  | 0 | |
|  | FP (n = 18) |  |  |  |  |  | |  |  |  |  | |
| **Central Bass Strait summer** | | | | | | | | | | | | |
|  |  | **2009** | | | **2010** | | | | **2011** | | | |
|  |  | LP  (n = 18) | STSW  (n = 20) | FP  (n = 0) | LP  (n = 19) | STSW  (n = 20) | | FP  (n = 6) | LP  (n = 10) | STSW  (n = 20) | FP  (n = 17) | |
| **2009** | LP (n = 18) |  | 0 | - | 7.9 | - | | - | 28.5 | - | - | |
|  | STSW (n = 20) |  |  | - | - | 45.1 | | - | - | 48.9 | - | |
|  | FP (n = 0) |  |  |  | - | - | | - | - | - | - | |
| **2010** | LP (n = 19) |  |  |  |  | 0 | | 11.5 |  | - | - | |
|  | STSW (n = 20) |  |  |  |  |  | | 0.1 | - | 18.7 | - | |
|  | FP (n = 6) |  |  |  |  |  | |  | - | - | 20.2 | |
| **2011** | LP (n = 10) |  |  |  |  |  | |  |  | 0 | 1.7 | |
|  | STSW (n = 20) |  |  |  |  |  | |  |  |  | 0 | |
|  | FP (n = 17) |  |  |  |  |  | |  |  |  |  | |
| **Eastern Bass Strait summer** | | | | | | | | | | | | |
|  |  | **2009** | | | **2010** | | | | **2011** | | | |
|  |  | LP  (n = 18) | STSW  (n = 20) | FP  (n = 0) | LP  (n = 20) | | STSW  (n = 20) | FP  (n = 0) | LP  (n = 10) | STSW  (n = 13) | | FP  (n = 0) |
| **2009** | LP (n = 18) |  | 0 | - | 23.1 | | - | - | 6.0 | - | | - |
|  | STSW (n = 20) |  |  | - | - | | 12.3 | - | - | 0 | | - |
|  | FP (n = 0) |  |  |  | - | | - | - | - | - | | - |
| **2010** | LP (n = 20) |  |  |  |  | | 0 | - | 14.8 | - | | - |
|  | STSW (n = 20) |  |  |  |  | |  | - | - | 21.4 | | - |
|  | FP (n = 0) |  |  |  |  | |  |  | - | - | | - |
| **2011** | LP (n = 10) |  |  |  |  | |  |  |  | 0 | | - |
|  | STSW (n = 13) |  |  |  |  | |  |  |  |  | | - |
|  | FP (n = 0) |  |  |  |  | |  |  |  |  | |  |
| **Western Bass Strait winter** | | | | | | | | | | | | |
|  |  | **2008** | | | **2009** | | | | **2010** | | | |
|  |  | LP  (n = 3) | FP  (n = 0) | CDP  (n = 0) | LP  (n = 2) | | FP  (n = 0) | CDP  (n = 0) | LP  (n = 16) | FP  (n = 18) | | CDP  (n = 8) |
| **2008** | LP (n = 3) |  | - | - | na | | - | - | na | - | | - |
|  | FP (n = 0) |  |  | - | - | | - | - | - | - | | - |
|  | CDP (n = 0) |  |  |  | - | | - | - | - | - | | - |
| **2009** | LP (n = 2) |  |  |  |  | | - | - | na | - | | - |
|  | FP (n = 0) |  |  |  |  | |  | - | - | - | | - |
|  | CDP (n = 0) |  |  |  |  | |  |  | - | - | | - |
| **2010** | LP (n = 16) |  |  |  |  | |  |  |  | 21.8 | | 0.5 |
|  | FP (n = 18) |  |  |  |  | |  |  |  |  | | 8.1 |
|  | CDP (n = 8) |  |  |  |  | |  |  |  |  | |  |
| **Central Bass Strait winter** | | | | | | | | | | | | |
|  |  | **2008** | | | **2009** | | | | **2010** | | | |
|  |  | LP  (n = 7) | FP  (n = 0) | CDP  (n = 10) | LP  (n = 20) | | FP  (n = 0) | CDP  (n = 4) | LP  (n = 20) | FP  (n = 3) | | CDP  (n = 15) |
| **2008** | LP (n = 7) |  | - | 0 | 2.7 | | - | - | 8.2 | - | | - |
|  | FP (n = 0) |  |  | - | - | | - | - | - | - | | - |
|  | CDP (n = 10) |  |  |  | - | | - | 0 | - | - | | 36.1 |
| **2009** | LP (n = 20) |  |  |  |  | | - | 0 | 15.0 | - | | - |
|  | FP (n = 0) |  |  |  |  | |  | - | - | - | | - |
|  | CDP (n = 4) |  |  |  |  | |  |  | - | - | | 0 |
| **2010** | LP (n = 20) |  |  |  |  | |  |  |  | na | | 0 |
|  | FP (n = 3) |  |  |  |  | |  |  |  |  | | na |
|  | CDP (n = 15) |  |  |  |  | |  |  |  |  | |  |

**Figure S1: Niche overlap - δ^13^C and δ^15^N values in whole blood of little penguins (winter and summer), short-tailed shearwaters (summer), fairy prions (winter and summer) and common diving petrels (winter): a) Western Bass strait, b) Central Bass Strait and c) Eastern Bass Strait, South-East Australia.** Solid lines represent the standard ellipses corrected for sample size (SEAc) (. The samples were collected in winter (July-August) 2008, 2009 and 2010, and summer (January-February) 2009, 2010 and 2011.


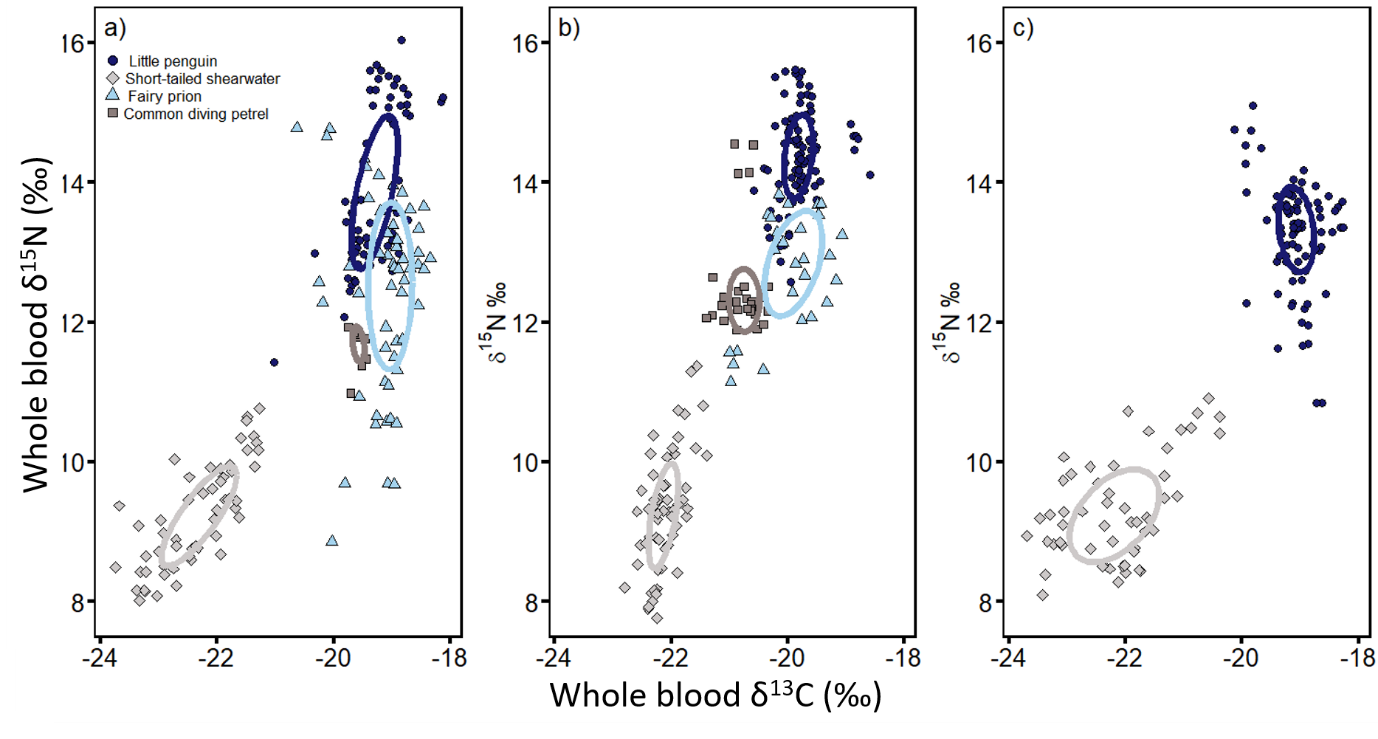

Supplement: Supplemental Information 1 [file peerj-08-8700-s001.docx]
